# Half-Yearly Reports of the Government Charitable Dispensaries Established in the Bengal and North-Western Provinces, from 1st August 1840, to 31st January 1842

**Published:** 1845-01

**Authors:** 


### Art. VII.

*Half-yearly Reports of the Government Charitable Dispensaries established in the Bengal and North-western Provinces, from* 1*st August* 1840, *to* 31*st January* 1842. *Printed by Authority.—Calcutta,* 1843, pp. 340.

It appears that, within the last few years, dispensaries have been established in the larger towns of Bengal and the North-western provinces by government, but assisted in some instances by private subscription. To several of these, small hospitals are attached. The Reports derive much of their interest from the circumstance that the medical duties of the institutions are performed principally by native sub-assistant surgeons, educated at the Medical College in Calcutta. Some of them have also native apprentices, of a respectable station in life.

In looking over the statistical documents transmitted from each dispensary, we observed that the greater proportion of the cases treated, are the primary or secondary effects of miasmata. At Dacca, the cholera raged violently in November and December 1840, and two thirds of those attacked died ; it was also extremely fatal at Benares, during the hot season of the same year. Of the whole number of 2005 cases treated at the dispensary during the half-year extending from August 1st to February 1st, 470 were cases of intermittent fever, 322 of splenitis, and 196 of cholera biliosa, diarrhea, and dysentery, amounting to 50 per cent. of all treated. The average time occupied in the treatment of intermittents was 22 days, of splenitis 29 days. From the report from Delhi, we learn that a great number of the male inhabitants of that city lose their virility at a comparatively early age, and apply at the dispensary for aphrodisiacs. Cases of gonorrhea, syphilis, and syphiloid diseases are extremely numerous among the poor of the provincies. Mr. Keane, assistant surgeon at Moorshedabad, states:

"Venereal affections are very common in all their forms. Gonorrhea and chancre are very prevalent. If taken in time, they are seldom intractable. Gonorrhea readily yields to cooling medicine and cubebs; but sometimes injections are necessary. Chancres have been treated with cooling medicines, charcoal poultices, simple dressing, and sometimes the internal exhibition of diluted nitric acid. Severe cases of phagedenic ulcers often occur, the whole of the penis being frequently destroyed at the time of admission, but in no instance has a cure failed to be effected by charcoal poultices, sulphate of copper lotions, simple dressing, and iodine and bark administered internally. Venereal disease is so prevalent, not only in the city, but even in retired country villages, that some general measure for its diminution or prevention, if such could be effected, could not fail to have a beneficial influence on the general health of the people." (p. 26.)

The morals of these people are not placed in a creditable light by the following statement, although, we believe, M. Ricord's experience at Paris is not very dissimilar. We quote sub-assistant surgeon Ishur Chander Gangoolee, of the Benares Dispensary:

"*Syphilis.* This is far the most common disease in Benares, and is frequently the result of unnatural intercourse. Out of 328 cases of primary syphilis, 80 were fungoid ulcers in the muco-integumentary membrane of the anal opening. The fungus yielded rapidly to powdered sulphate of copper and exposure to the air, and to the internal use of calomel. Seventeen cases of phymosis were operated upon; of 170 patients suffering from secondary syphilis, 37 were young children who had acquired it from their parents." (p. 238.)

Bronchocele, elephantiasis and various forms of leprosy are frequent in some of the districts; at Patna, for example,

"The *elephantiasis* cases are many of them very severe, and not unfrequently are seen attacking the scrotum, so as to produce tumours of considerable magnitude; the disease begins with some pain in the inguinal glands, and it is probable that, if this were attended to in the early stage, the morbid action which is afterwards set up might be prevented; but the natives take little notice of slight ailments, and it is not until diseases assume a formidable appearance, that they seek relief. I have little doubt but that local bleeding in the first instance and free purging with rest would allay the first symptoms, but they are not employed; and we see men and women too (though not so frequently,) going about the streets of Patna with legs that would literally bear comparison with those of the elephant, from which the disease derives its name. I have dissected limbs of this description, and found the disease entirely confined to the skin and cellular membranes, including the fascia, which are all conglomerated into a fatty gristly substance, with little vascularity, the subjacent muscles being paler and smaller than usual.

"The *leprosy* is a very common disease at Patna amongst the lower classes, and occasionally finds its way among the respectable families; it is often complicated with a venereal taint, in which case the disease rapidly progresses, the joints of the extremities ulcerate, the bones of the nose are absorbed, the skin thickens and becomes blotched, and the whole body is soon reduced to a pitiable state of wretchedness. Some hundreds of such objects wander about the streets of Patna, and being outcasts from their families, depend for their very existence on the precarious charity of the benevolent; the disease is more common among the Hindoo than the Mussulman population; but it attacks both, and the greater frequency in the one case than in the other may arise from food and habits of life." (p. 51.)

Burs or suffadee, and the uprus, are two other cutaneous diseases common in this district, and connected, as well as the syphilitic affections, with the uncleanliness of the natives. It would be well if they had a Mosaic legislator among them, or that a prophet should rise up to preach frequent ablutions.

Ram Eshur Awushtee, the sub-assistant surgeon at Patna, attributes the frequency of bronchocele to the impurity of the water. It occurs principally among the lower castes. The treatment he has found most successful is to tie the neck with a towel for three or four months, and to rub the following ointment over the whole surface of the tumour twice a day. We copy the prescription verbatim:

"℞ Sujjee communis, ℥j.

Adeps præparatæ, ℥j.

Ol. lini, ℥iss.

Fiat unguent id illineat ægra supra tumorem bis in dies."

Hepatitis seems rare, and so also phthisis.

Although native medicines are used as much as possible, the medical treatment at these dispensaries seems to be conducted after the European model. The old classification, too, of Cullen greets our eyes, reminding us of our scholastic labours. At Benares, "in many hundred cases of cholera," Mr. Butler states,

"Life was saved by the distribution throughout the city and district of pills composed of two grains of opium and four grains of calomel, with instructions for their administration, and for the use of leeches and aperients in cases in which these remedies might be required; I had the satisfaction of hearing from the officiating magistrate that these means were in general remarkably successful. I had an opportunity of seeing the disease in its most malignant form at the residence of the ex-Raja of Coorg, where two thirds of the cases proved fatal, as might have been expected from the miasmatous nature of the locality; and where many cases of intermittent fever of an intractable character subsequently occurred. There was no other peculiarity in the fever of the year; it was, as usual, accompanied in some cases by severe symptoms and followed by enlargement of the spleen." (p. 11.)

Mr. Keane observes on diseases of the spleen:

"Even when there is little perceptible enlargement, the patient may be distinguished by a look of debility; and on everting the eyelids, the conjunctiva is found very pale. The spleen sometimes attains an enormous size, and in all cases of enlargement the least violence as a fall, blow, &c., will cause its rupture and the immediate death of the patients. Enlarged spleen is very generally a consequence of repeated attacks of fever. The treatment generally pursued is to give tonics combined with purgatives, assisted by leeches and blisters; unless the disease be complicated with other organic derangements this plan almost never fails. Tobacco plasters have been sometimes tried in recent cases, applied over the region of the spleen, and have effected very sudden cures." (p. 27.)

Mr. Bacon, of the Mooradabad Dispensary, has used the root of the Madar plant with great advantage in old ulcers of long standing, in many varieties of leprosy, in syphilis, scrofula and cutaneous eruptions. Ramnarain Doss also, of the Cawnpore Dispensary, relates a case of lepra in which it was successfully used. Mr. Bacon observes:

"With an adult I generally begin with three grains three times a day. If this quantity does not produce any disorder of the stomach, the dose is generally increased. In many incipient cases of lepra, the benefical effecits of this medicine were evident. After a lapse of a week or ten days, the pain is alleviated and the itching less. The skin becomes soft, and its discoloration gradually disappears. Inherpes, many of them cases of some years' standing, the use of the root has been most successful. In syphilitic ulcers, I have not depended on it alone, but have combined it with either the blue pill or Plummer's pill; and with this combination I do not think the constitution suffers so much, as in a protracted use of mercury alone it invariably does. In the shape of ointment I have used it with the best effect in bad conditioned sores." (p. 179.)

A native remedy for asthma is subjoined in the words of the reporter, Ram Eshur Awasthee:

"Compound Powder of Cauler Zeeree. This powder has been given with great success in cases of asthma and cough, when other medicines, such as compound squill pills, ipecacuanha, &c., have failed in affording any relief to the patients. The powder stated above is composed of the following ingredients:

"Take of Powder of Calee Zeeree, gr. x.
Sellageet, Puthanee, Loadh, aa gr. iv.
Mix and give three times a day."
(p. 214.)

Amongst the surgical cases we find several instances of capital operations successfully performed. Umachurn Set supplied a man with a nose after the Taliacotian method, who had had his own cut off in a quarrel. Numerous cases of hydrocele were treated successfully with iodine injections. We extract the following report by the operator:

"*A case of lithotomy,* operated by Ramnarain Doss, sub-assistant surgeon. Gobiadah, a Bramhin lad, aged 7, admitted on the 7th July 1841, for stone in the bladder; his father states that the boy has been labouring under this disease for two years, and says, makes water with great difficulty; he has always been seen to squeeze his penis; urine drops involuntarily; and he sleeps very rarely at night: the urine examined to ascertain the state of the bladder, and urine is found to contain pus in it; and therefore it seems that the bladder is diseased; the bladder examined by catheter, and the stone is found to be a large one.

"To have anodyne draught at night.

"10th July. Says had no sleep last night. To have ol. ricini, ℥j, immediately.

"Had three free stools at twelve o'clock, and to have no food. Tinct. opii administered, so as to make him retain urine in order to facilitate the operation.

"10th July, two o'clock. The stone extracted by the lateral operation; the operation lasted only five minutes, while extracting, the stone, being soft in its nature, broke into halves, and into small pieces, as it is seen in the drawing. Besides these, sand-like grains were found lying on the surface of the bladder, therefore to wash them down cold water has been injected. After the operation, a female catheter was kept in the bladder on purpose to let the urine out; a few minutes after, the boy was seen to sleep very profoundly, without any opiates after the operation; and his father said that he never saw him to sleep so very profoundly within these two years.

"13th July. The catheter taken out, complains of pain on pressure on the pubic region; skin little warm; tongue foul; says had no stool since yesterday; the lower part of the belly is to be fomented with hot water.

"To have ol. ricini, ℥j, immediately. As for diet, to have only sago with milk, &c." (p. 256.)

And so the case went on to a cure, like all others of the kind. Ramnarain Doss states: "all the lithotomy operations that have been performed in this dispensary were successful, and all the cases got well without any dangerous symptoms, as peritonitis and others, and we are proud to say that we have not lost a single patient in this operation."

Shumachum Dutt, sub-assistant surgeon at Allahabad, (and subsequently at Jubbulpore,) and joint-editor with Dr. Spilsbury of the Hindostanee edition of the London Pharmacopœia, performed the following operations successfully: Amputation below the knee 1; of the penis 1; for cataract 4; for fistula in ano 3; fistula in perineo 1; paracentesis of the abdomen 8; for entropium 3; for removal of encysted tumours 2. In medico-chirurgical cases we find the treatment of these young native practitioners judicious. Let the following case, reported by Ramnarain Doss, be our witness:

"*A case of concussion of the brain.* Buddoo, a barber by occupation, aged 17, admitted 9th May 1841, for concussion of the brain. It is stated that, in the bazaar, he had been kicked down by a horse; on admission he was as if in profound sleep, and does not answer to the questions put to him; the pulse was small and slow; the head examined, no fracture and nothing of the kind was found, but a small cut on the forehead; at eight o'clock the pulse rose, and began to be full, strong, and hard; and the skin became warm which was before of natural temperature; at nine o'clock at night he was bled to one lb.; after the bleeding he began to speak, but not rationally. He was asked where is he, and what is the matter with him, (he answered after a long time,) that he is in the Kuttowal's Chowbutarah, the place where he was first taken on the reception of the injury, and that nothing is the matter with him; the head is to be kept cool by cold lotion, and to have hydrarg. submur., gr. ix, immediately.

"10th May. Pulse quick and weak; skin little warm; and still says that he is in the Kuttowal's Chowbutarah, and if asked what is his name, he says Buddoo, a barber by occupation; had no stool last night; to have pulv. jalap. co., ʒj, immediately.

"At twelve o'clock he had one stool, and therefore to have sulph. magnes., ℥j, immediately.

"10th May, four o'clock. At four o'clock he had three free stools; pulse full and hard; and if questioned answers rationally; complains of pain in the head, and round the wound; skin too much warm; at five o'clock he became again insensible; pulse became fuller, stronger, and harder than before; and when asked where is he and what is the matter with him, he still says that he is in the Kuttowal's Chowbutarah, and that nothing is the matter with him.

"Bled again to ten ounces; to have hydrarg. submur., gr. iv, pulv. antimonialis, gr. iv, one time in the night.

"11th May. Bowels once moved very early this morning; now he speaks very rationally, and says he is doing well; to have pulv. jalap. co., ʒj, immediately." (p. 248.)

And he did well.

Amongst the documents published in this volume is a sketch of the medical topography (called a Statistical Report) of Patna, by Dr. Davies. There are hospitals for the prisoners, the insane, and the poor, the latter having thirty beds. Appended to this Report is an account of the native treatment of leprosy and elephantiasis in Patna, and the formulæ of the medicines in use; also a table of indigenous medicines, with their Persian and Latin or English synonym; their native name in Roman and Hindostanee character; and their operation, use, and doses in English. Both these documents, as well as the Report of the Patna Dispensary, are written by Ram Eshur Awushtee.

The object of the government in educating natives as practitioners, seems to be to supply the population of India with efficient medical advice, and with efficient indigenous remedies. The Hakims or native doctors have no knowledge whatever of anatomy or physiology, not even of the circulation of the blood. Dr. Davies says, "the state of popular medicine differs little from that which has existed for many centuries; the catalogue of materia medica has been greatly added to; but no knowledge of the structure of the human body exists among the best informed of the native physicians; and, as far as I can ascertain, the doctrine of the dogmatists and empirics who lived two or three centuries before Christ, and retained the mode of practice of Hippocrates, but pretended to despise his reasoning, are those adopted in the present day." These regularly educated practitioners are therefore permitted to engage in private practice. In doing this they have, *cæteris paribus*, the same difficulties to overcome as the young practitioner in Europe. The old Hakims are jealous of them, (old Hakims are jealous in England,) and do all in their power to thwart them. Mr. Ross, the surgeon, at Delhi, says, the Mussulman physicians are numerous there, many being dignified with the title of court-physicians, and having extensive and lucrative practice. These individuals do not hesitate to libel their more learned and energetic neighbours, by circulating reports that they use different parts of the human body in the composition of their medicines; and they have made many efforts to prevent patients going to them, even setting men to ply about the door of the dispensary, and dissuade the patients from entering. This opposition seems to have discouraged some of these young men. Amachurn Set, sub-assistant surgeon at Agra, in the proscript to his report, states:

"My private practice, I am sorry to say, is rather limited, and does not remunerate me sufficiently for my troubles. I have a little practice among the writers in the public offices, both the East Indians and the Bengalies; and I am also sometimes called in by the natives of the place in serious cases, after the Hakeems have failed in affording any relief. I am occasionally rewarded by the wealthy natives on the favorable termination of the cases I am called on to attend." (p. 290.)

It is manifest, however, that these young men are winning golden opinions. The dispensary at Dacca was visited by some native gentlemen, visiting members of the committee, and the following observations recorded by them in the visitors' book:

"It was crowded with patients, and Baboo Nobinchunder Paul was busy distributing medicines with zeal and activity, so that the inhabitants, being pleased with his services, gave him sincere thanks. They say, that by his services, several patients have recovered, and we also perceive that some men belonging to us have been cured by him." (p. 201.)

The Rev. Mr. Sheppard, the chaplain at Dacca, bears high testimony to the character of this young native surgeon, by stating that, in his opinion, Nobinchunder Paul "is an efficient and accomplished practitioner. His kindness, attention, and professional success in the treatment of diseases have gained the confidence of the inhabitants; and to these excellent qualities and his good conduct in every respect, I have no hesitation in ascribing the progress which the institution is daily making in popularity and usefulness." Mr. Cumberland, the assistant-surgeon at Porree, intrusted himself with confidence to the care of his sub-assistant Baboo Nilmoney Dutt, when labouring under an attack of bilious remittent fever, and has every reason "to be satisfied with his prompt and judicious treatment." At Allahabad, the principal native doctor condescended to place his own son under the care of Shamachurn Dutt for fistula in ano. We observe, too, that Jaudub Chander Set, of the Bareilly Dispensary, has nothing to complain of, and states gallantly that "patients are daily increasing in number; natives, both rich and poor, seem to be pleased with me, because they get good medicines and good treatment from me; the Hakeems of the town are jealous of me, because they have lost much of their practice; the principal operations that I have performed are amputation, lithotomy, and several operations on the eye." (p. 272.)

But in India, as in Europe, science has to contend with the credulity of the ignorant people as well as with the selfishness of the ignorant practitioner. There, a supposed incarnation of a Hindoo divinity is as attractive to the mob as the shrine of a water-doctor in England. In Moorshedabad, one cause for a diminution of dispensary patients was this: "In the extremity of the Zillah to the eastward, there was believed to have arisen an incarnation of a Hindoo divinity, which drew off immense crowds daily, in the hope of being relieved of all sorts of ailments curable and incurable." (p. 24.)

We are quite sure this volume of Reports on the government dispensaries in Bengal will be highly satisfactory to their promoters, and to the teachers of the young native surgeons attached. Not the least remarkable circumstance connected with their education, is the proficiency in the English language displayed by them in these reports.

---

